# Answer-locked ERP dynamics during arithmetic verification in children with developmental dyscalculia

**DOI:** 10.3389/fpsyt.2026.1877000

**Published:** 2026-08-27

**Authors:** Qinfen Zhang, Shiyan Ji, Yuhao Wang, Rui Wang

**Affiliations:** Children’s Health Research Center, Changzhou Children’s Hospital of Nantong University, Changzhou, Jiangsu, China

**Keywords:** arithmetic processing, arithmetic verification, developmental dyscalculia, ERPs, school-age children

## Abstract

Children with developmental dyscalculia (DD) show persistent difficulties in arithmetic, yet the temporal characteristics of neural processing after the presentation of a proposed answer remain insufficiently understood. This study used an answer-locked event-related potential (ERP) design to examine neural dynamics during arithmetic verification in 76 children aged 8–12 years, including 37 children with DD and 39 typically developing (TD) children. Participants completed addition, subtraction, and multiplication verification problems while electroencephalography was recorded. Peak amplitude and peak latency of the N2a, N2b, and P3 components were measured at Fz and Cz. Component-specific analyses were complemented by omnibus models across ERP components and electrodes, and Holm correction was applied to control for multiple comparisons. Children with DD showed substantially lower accuracy and longer reaction times than TD children. After correction, the DD group showed longer N2a peak latencies at Fz and Cz, longer N2b peak latency at Fz, and smaller P3 peak amplitude at Fz. The surviving ERP effects were moderate to large in statistical magnitude, and omnibus analyses further indicated that the pattern of group differences varied across components and electrodes. By time-locking neural activity to proposed-answer onset, these findings delineate a robust pattern of delayed N2 responses and reduced later frontal P3 amplitude in DD, clarifying when group differences emerge during arithmetic verification. These findings characterize group-level differences in answer-related neural processing but do not isolate the contributions of calculation and verification-related demands.

## 1 Introduction

Developmental dyscalculia (DD) is a neurodevelopmental learning disorder characterized by persistent difficulty in acquiring arithmetic skills despite adequate intelligence, educational opportunity, and sensory function. Children with DD often show weaknesses in numerical magnitude processing, symbolic number mapping, arithmetic fact retrieval, and calculation fluency (1–3). These number-specific accounts address core arithmetic difficulties, whereas arithmetic verification additionally requires the temporary maintenance, comparison, and evaluation of numerical information.

Arithmetic verification is a composite task that places demands on both arithmetic processing and broader cognitive functions. When a child sees a proposed answer, the task is not simply to calculate. The child must hold the arithmetic expression in mind, generate or retrieve an expected result, compare that result with the presented answer, evaluate their correspondence, and select a response. Performance therefore reflects the combined contributions of arithmetic competence, working memory, attentional regulation, answer comparison, and response selection. Behavioral studies have shown that executive functions are closely associated with children’s mathematical achievement, and that inhibition, shifting, and working memory contribute to individual differences in arithmetic performance (4–6). Children with arithmetic learning difficulties also show weaknesses in inhibition and cognitive flexibility, suggesting that poor mathematical performance may partly reflect inefficient control over task-relevant information (7).

Evidence from DD further supports this broader view. Children with DD have been reported to show poorer performance on inhibitory-control tasks (8). Other work suggests that DD is linked not only to number-specific deficits but also to weaknesses in visuospatial memory and inhibitory-control performance (9). Working memory is also consistently related to mathematics achievement, with the strength of this association depending on the domain of working memory and the type of mathematical skill being assessed (10). These findings suggest that domain-general cognitive abilities may contribute to arithmetic verification, although their contribution cannot be separated from calculation difficulty in the present task.

Event-related potentials (ERPs) provide a useful way to examine the temporal dynamics of neural processing during arithmetic verification. The N2 component has been linked to conflict monitoring, mismatch detection, and response selection, especially over fronto-central scalp sites (11–13). The P3 component is usually associated with attentional resource allocation, stimulus evaluation, and context updating (14, 15). In an arithmetic verification task, these components can help characterize the timing and magnitude of answer-related comparison and evaluation, although they are not process-specific markers of inhibition or cognitive control. These functional associations are task dependent, and findings from tightly controlled adult conflict paradigms cannot be assumed to have the same functional significance in a child arithmetic-verification task.

Previous ERP studies have shown that arithmetic processing is sensitive to task difficulty, problem size, and verification demands (16). ERP studies involving children with DD have also reported atypical neural responses during arithmetic tasks, suggesting alterations in the timing and magnitude of arithmetic-related processing (17). Behavioral work has documented poorer performance on inhibitory-control tasks in children with DD (9), and Chao et al. (18) reported atypical ERP responses during numerical tasks involving response-control demands. Thus, the present study does not seek to establish inhibitory difficulty as a previously unrecognized feature of DD. Rather, its contribution is to characterize the timing of neural responses specifically locked to proposed-answer onset during arithmetic verification. It remains unclear whether group differences in this answer-locked activity emerge during earlier or later post-answer periods, or both.

The present study used an answer-locked ERP design to examine neural processing during arithmetic verification in children with DD. Unlike operation-locked analyses that focus on the processing of arithmetic expressions, the present analysis focused on neural responses after the proposed answer appeared. We expected children with DD to show lower accuracy and longer reaction times than TD children. Because fronto-central N2 activity has been associated with mismatch detection, conflict-related processing, and response selection, we predicted that the DD group would exhibit prolonged N2a and N2b latencies and reduced (less negative) N2a and N2b amplitudes, reflecting delayed and attenuated answer-related processing. Based on the association of P3 with attentional resource allocation, stimulus evaluation, and context updating, we further predicted that children with DD would show a smaller P3 amplitude and a longer P3 latency, reflecting altered later answer-related processing. These hypotheses concern neural processing within a composite arithmetic-verification task and do not assume that the observed differences reflect an isolated deficit in inhibition or cognitive control.

## 2 Methods

### 2.1 Participants

Seventy-six children aged 8–12 years were included in the final analysis, including 37 children with DD and 39 typically developing (TD) controls. Participants were recruited from a pediatric outpatient clinic and local primary schools. All children were right-handed and had normal or corrected-to-normal vision and hearing.

Children in the DD group met the following criteria: (1) age 8–12 years; (2) an age-normed score of at least 80 on the Chinese version of Raven’s Standard Progressive Matrices, to exclude marked nonverbal intellectual impairment; (3) an overall score on the Chinese Basic Mathematical Ability Test for Primary School Students at least 1 standard deviation below the norm for the child’s current grade (Z ≤ −1.0); (4) a converted T score below 70 on the Dyslexia Checklist for Chinese Children, indicating a score below the screening cutoff for suspected dyslexia; (5) a score below 65 on the Chinese revision of the Pupil Rating Scale–Revised: Screening for Learning Disabilities, together with teacher confirmation that the mathematical difficulties had persisted for at least 12 months; (6) access to adequate educational opportunities; and (7) right-handedness. Children were excluded if they had a severe reading or writing disorder; a current or previous clinical diagnosis of attention-deficit/hyperactivity disorder or autism spectrum disorder; neurological disease or brain injury; uncorrected visual or hearing impairment; marked emotional disturbance; or inadequate educational exposure. The Z ≤ −1.0 threshold was therefore applied as part of a multistep identification procedure that also considered persistent functional difficulty, educational exposure, nonverbal intellectual ability, and relevant exclusion criteria, rather than as the sole criterion for DD.

Raven’s Standard Progressive Matrices contains 60 nonverbal matrix-reasoning items. One point is awarded for each correct response, and the raw total is converted according to age-based Chinese norms (19). The Chinese Basic Mathematical Ability Test for Primary School Students assesses mathematical calculation as well as logical-reasoning and visuospatial abilities. Scores are interpreted using grade-specific norms, and an overall score below 1 standard deviation from the grade mean was used as the mathematical-performance criterion for DD (20, 21). The scale has demonstrated satisfactory psychometric properties in Chinese primary-school samples, with subtest Cronbach’s α coefficients above 0.70, split-half reliability of 0.83, and a Spearman–Brown corrected reliability coefficient of 0.90. Most subtest test–retest correlations exceeded 0.70. Factor loadings were above 0.70, subscale–total correlations exceeded 0.50, and the scale showed significant discrimination between higher- and lower-performing groups (*P* < 0.01) (21).

Reading difficulties were screened using the Dyslexia Checklist for Chinese Children (DCCC), a 55-item parent-report instrument covering eight domains of Chinese reading-related difficulty. Items are rated on a five-point frequency scale, with higher scores indicating greater reading difficulty, and raw scores are converted to standardized T scores (22). In the present study, a T score of 70 or above was considered a positive screen for suspected dyslexia; therefore, only children with T scores below 70 were included. The DCCC has demonstrated satisfactory internal consistency, test–retest reliability, and construct validity.

The Chinese revision of the Pupil Rating Scale–Revised is a 24-item teacher-rated screening measure covering five functional domains. Items are rated on a five-point scale, with higher scores indicating stronger functioning. A total score below 65 was used as the screening criterion for learning difficulties (23).

TD children had mathematical performance at or above the average for their grade and a score of 65 or higher on the Chinese revision of the Pupil Rating Scale–Revised. They met the same Raven, reading-screening, educational-opportunity, handedness, and exclusion criteria as the DD group.

General anxiety and depressive symptoms were assessed at enrollment using the Zung Self-Rating Anxiety Scale (SAS) and the Zung Self-Rating Depression Scale (SDS), administered through the Shanghai Huicheng Child Psychological Assessment Software 2.0 (HC-ET-XL-DJ-2.0). All participants were classified as being within the non-clinical range. Age-standardized Working Memory Index (WMI) and Processing Speed Index (PSI) scores from the Chinese version of the Wechsler Intelligence Scale for Children–Fourth Edition (WISC-IV) were available for all participants as part of a broader cognitive assessment. These indices were not used as eligibility criteria or for group assignment but were included in supplementary sensitivity analyses.

Family socioeconomic status (FSES) was calculated as a composite score based on parental education, parental occupation, and household income. Higher FSES scores indicated higher family socioeconomic status. FSES was used to compare whether the DD and TD groups were comparable in family background characteristics at baseline.

The study was approved by the institutional ethics committee. Written informed consent was obtained from parents or legal guardians, and verbal assent was obtained from all children before testing.

### 2.2 Arithmetic verification task

Participants completed an arithmetic verification task consisting of addition, subtraction, and multiplication problems within 20. Each trial presented an arithmetic expression followed by a proposed answer. Children were instructed to judge whether the proposed answer was correct or incorrect by pressing one of two response keys.

The task included 150 trials, with 50 trials for each operation type. Correct and incorrect answers were balanced across trials. The order of trials was randomized for each participant.

Each trial began with a fixation stimulus presented for 600 ms. The first operand, arithmetic operator, and second operand were then presented sequentially for 300 ms each, with a 500-ms blank interstimulus interval between the first operand and the operator and between the operator and the second operand. After the second operand disappeared, a blank interval of 2000 ms was presented before the proposed answer appeared. The proposed answer remained on the screen for up to 2000 ms or until a response was made. Participants judged whether the proposed answer was correct or incorrect by pressing one of two response keys, with the right key assigned to correct answers and the left key assigned to incorrect answers. After the response, a blank screen was presented for 3000 ms before the next trial began. Before the formal experiment, participants completed a criterion-based practice session using arithmetic problems that were not included in the main task. The number of practice trials was not fixed because children differed in the amount of practice needed to understand the procedure. Instructions and practice trials were repeated when necessary, and participants proceeded to the formal experiment only after they could correctly explain the response-key mapping and perform the task appropriately under the experimenter’s observation.

**Figure 1:**
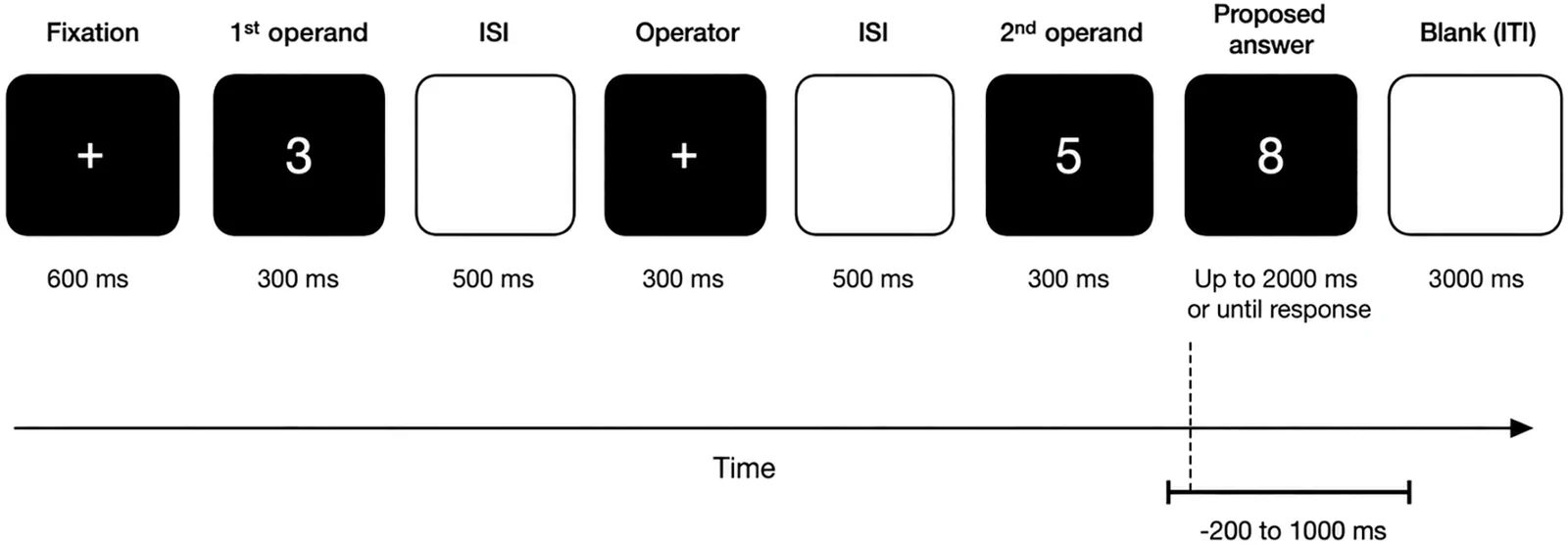
Timeline of the arithmetic verification task. Each trial included a 600-ms fixation, three arithmetic elements presented for 300 ms each with 500-ms ISIs, a 2000-ms blank interval, and a proposed answer displayed for up to 2000 ms or until response. A 3000-ms blank screen followed. ERPs were time-locked to answer onset. ISI, interstimulus interval; ITI, inter-trial interval.

For the present study, the main ERP analysis focused on neural responses time-locked to the onset of the proposed answer. This answer-locked design was used to characterize neural processing following the onset of the proposed answer. The task procedure and answer-locked epoching scheme are illustrated in **Figure 1**.

### 2.3 EEG recording

EEG was recorded using a 32-channel Brain Products system according to the international 10–20 electrode placement system. Participants were seated approximately 80 cm from the monitor in a quiet, dimly lit room. Stimuli were presented centrally using E-Prime software. EEG signals were sampled at 1000 Hz during acquisition, and electrode impedance was kept below 5 kΩ throughout the recording. The online reference was placed at FCz. Horizontal and vertical electrooculograms were recorded to monitor eye movements and blinks.

### 2.4 EEG preprocessing

EEG data were processed in MATLAB using EEGLAB and ERPLAB toolboxes (24, 25). Continuous EEG data were band-pass filtered from 0.1 to 35 Hz to remove slow drift and high-frequency noise, and were then down-sampled to 500 Hz for subsequent analysis. For the answer-locked ERP analysis, epochs were extracted from −200 to 1000 ms relative to the onset of the proposed answer. The −200 to 0 ms interval was used for baseline correction.

Artifacts related to eye movements, blinks, muscle activity, and nonphysiological noise were identified using visual inspection and independent component analysis. Epochs with voltage fluctuations exceeding ±100 μV were rejected. The data were then re-referenced to the average reference. Bad channels were interpolated when necessary using information from the remaining valid channels. In the DD group, the mean number of interpolated channels was 1.30 ± 1.05 (range: 0–4), compared with 1.10 ± 0.91 (range: 0–3) in the TD group. After ocular correction and artifact rejection, the mean percentage of retained answer-locked trials was 90.2 ± 3.5% in the DD group and 91.1 ± 3.8% in the TD group. For correct proposed-answer trials, the retained percentages were 90.8% and 91.7%, respectively; for incorrect proposed-answer trials, they were 89.6% and 90.5%, respectively. Only datasets retaining at least 80% of the required answer-locked trials after preprocessing were included in the statistical analyses. No participant was excluded solely for failing this criterion.

### 2.5 ERP measures

ERP analyses focused on N2a, N2b, and P3 at Fz and Cz, selected based on previous ERP literature and the fronto-central distribution of the collapsed grand-average waveforms. These components were examined to characterize the temporal profile of answer-related processing. Peak amplitude and latency were measured within component-specific windows applied consistently across groups and proposed-answer conditions and were extracted separately for correct and incorrect proposed-answer trials. Because the present task did not independently manipulate inhibition, conflict, or monitoring demands, these components were not treated as process-specific markers of any single cognitive operation.

N2a was defined as the most negative peak between 260 and 390 ms after proposed-answer onset, N2b as the subsequent negative peak between 400 and 550 ms, and P3 as the largest positive peak between 650 and 950 ms. Peak amplitude was defined as the voltage at the identified peak, and peak latency as the interval from proposed-answer onset to that peak. The measurement windows were defined before group-level statistical testing on the basis of previous ERP literature and the morphology of collapsed grand-average waveforms pooled across groups and proposed-answer conditions (12, 14–17). Thus, the windows were data-informed rather than strictly *a priori*, but they were selected without reference to group or condition differences and were applied identically to all participants and conditions. The labels N2a and N2b were used operationally to distinguish the earlier and later negative peaks.

### 2.6 Behavioral measures

Behavioral performance was assessed using accuracy and reaction time. Accuracy was calculated as the proportion of correct responses. Reaction time was defined as the interval between the onset of the proposed answer and the participant’s keypress. Reaction-time analyses included only correct-response trials. Responses faster than 300 ms were considered implausibly fast and were excluded. Trials with no response or responses exceeding the 2000-ms response window were also excluded. Behavioral indices were calculated for the overall task and separately for each arithmetic operation.

### 2.7 Statistical analysis

Statistical analyses were performed using SPSS version 27.0. Group differences in demographic variables were examined using independent-samples t tests for continuous variables and chi-square tests for categorical variables.

Accuracy scores were compared between the DD and TD groups using Mann–Whitney U tests and are reported as medians and interquartile ranges. Reaction times are reported as means and standard deviations. Homogeneity of variance was assessed using Levene’s test, and Welch’s t tests were used when the assumption of equal variances was violated. For each ERP component, peak amplitude and peak latency were analyzed separately using a 2 × 2 mixed-design ANOVA, with Group (DD and TD) as the between-subject factor and Answer Type (correct and incorrect proposed answers) as the within-subject factor. Separate ANOVAs were conducted for Fz and Cz. The main effects of Group and Answer Type and the Group × Answer Type interaction were examined.

To examine whether the pattern of group differences varied across ERP components, supplementary omnibus mixed-design ANOVAs were conducted separately for peak amplitude and peak latency, with Group as the between-subject factor and Component (N2a, N2b, and P3), Electrode (Fz and Cz), and Answer Type as within-subject factors. The Group × Component and Group × Component × Electrode interactions were of primary interest in these omnibus analyses. Greenhouse–Geisser corrections were applied to effects involving Component when the assumption of sphericity was violated.

The component- and electrode-specific 2 × 2 ANOVAs were retained as follow-up analyses. To control the family-wise error rate, Holm corrections were applied separately to three prespecified families of tests: the 12 Group main effects, the 12 Answer Type main effects, and the 12 Group × Answer Type interactions. Statistical interpretation of the component-specific analyses was based on the Holm-adjusted p values. Bonferroni-adjusted pairwise comparisons were performed where appropriate, and partial eta squared was reported as the effect-size estimate.

Group differences in the WISC-IV Working Memory Index (WMI) and Processing Speed Index (PSI) were examined using independent-samples t tests or Welch’s t tests, as appropriate, with Cohen’s d reported as the effect-size measure. Supplementary sensitivity analyses were conducted using 2 × 2 mixed-design ANCOVAs in which WMI and PSI were entered simultaneously as covariates. These analyses retained Group as the between-subject factor and Answer Type as the within-subject factor. The homogeneity-of-regression-slopes assumption was examined by testing the Group × WMI and Group × PSI interactions.

Pearson correlation analyses were used to examine associations between ERP latency indices and behavioral reaction time. Correlations were first performed across the full sample and then separately within the DD and TD groups. All tests were two-tailed, with the significance threshold set at *P* < 0.05.

## 3 Results

### 3.1 Participant characteristics

The final sample included 76 children, with 37 children in the DD group and 39 children in the TD group. The two groups did not differ significantly in age, sex distribution, nonverbal intelligence, or family socioeconomic status (all *P* > 0.05; **Table 1**). The groups also did not differ significantly in either the WISC-IV Working Memory Index (WMI) or Processing Speed Index (PSI).

**Table 1:** Participant characteristics.

Characteristic	TD group (n = 39)	DD group (n = 37)	χ²/t	*P* value
Sex (male/female)	27/12	29/8	0.819	0.365
Age (years)	9.01 ± 0.58	8.84 ± 0.81	1.096	0.277
Raven’s score	105.79 ± 12.23	104.32 ± 13.10	0.442	0.660
FSES	32.84 ± 0.65	32.63 ± 0.94	1.101	0.274
WISC-IV WMI	100.67 ± 9.84	100.54 ± 10.92	0.053	0.958
WISC-IV PSI	100.33 ± 10.04	99.76 ± 9.63	0.255	0.799

Continuous variables are presented as mean ± standard deviation. TD, typically developing; DD, developmental dyscalculia; FSES, Family Socioeconomic Status; WMI, Working Memory Index; PSI, Processing Speed Index.

### 3.2 Behavioral performance

Children with DD showed poorer performance than TD children during the arithmetic verification task (**Table 2**). Mann–Whitney U tests showed lower accuracy in the DD group for addition, subtraction, multiplication, and the overall task, whereas Welch’s t tests showed longer reaction times in the DD group for all three operations and the overall task (all *P* < 0.001). At the overall-task level, median accuracy was 0.22 lower and mean reaction time was 333.82 ms longer in the DD group than in the TD group. The corresponding standardized effect sizes were r_rb = −0.904 for accuracy and Hedges’ g = 1.244 for reaction time.

**Table 2:** Behavioral performance of TD and DD children during arithmetic verification.

Operation	Measure	TD group (n = 39)	DD group (n = 37)	Test statistic	*P* value	Effect size (95% CI)
Addition	Accuracy	0.94 (0.90, 0.98)	0.67 (0.55, 0.77)	*Z* = -6.623	< 0.001	-0.882 (-0.960, - 0.774)
	Reaction time (ms)	967.39 ± 203.62	1292.21 ± 358.14	T(56.44) = 4.826	< 0.001	1.111 (0.666, 1.655)
Subtraction	Accuracy	0.92 (0.88, 0.99)	0.63 (0.46, 0.76)	*Z* = -6.558	< 0.001	-0.873 (-0.971, -0.747)
	Reaction time (ms)	983.73 ± 192.18	1355.07 ± 348.77	T(55.37) = 5.706	< 0.001	1.315 (0.843, 1.942)
Multiplication	Accuracy	0.96 (0.92, 0.98)	0.79 (0.72, 0.84)	*Z* = -5.934	< 0.001	-0.834 (-0.946, -0.685)
	Reaction time (ms)	929.02 ± 150.58	1257.20 ± 320.78	T(38.81) = 5.182	< 0.001	1.354 (0.818, 2.098)
Overall task	Accuracy	0.95 (0.91, 0.97)	0.73 (0.54, 0.76)	*Z* = -6.779	< 0.001	-0.904 (-0.976, -0.804)
	Reaction time (ms)	975.96 ± 182.47	1309.78 ± 331.37	T(55.35) = 5.400	< 0.001	1.244 (0.779, 1.826)

Accuracy is presented as median (interquartile range) and was compared using Mann–Whitney U tests, with standardized Z values reported. Reaction time is presented as mean ± standard deviation and was compared using Welch’s t tests because of unequal group variances. TD, typically developing; DD, developmental dyscalculia.

### 3.3 Answer-locked ERP responses

**Figure 2:**
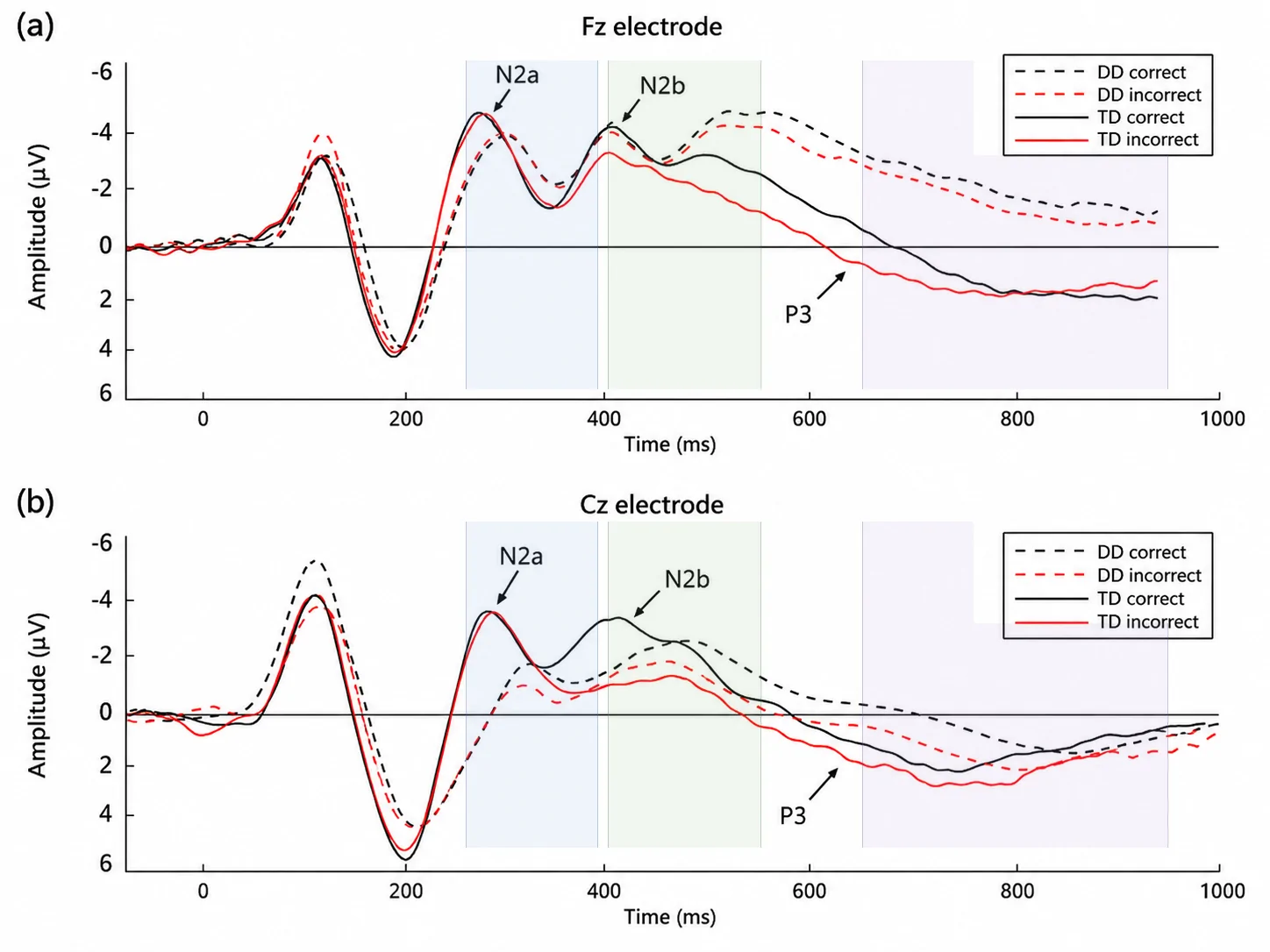
Grand-average answer-locked ERP waveforms at Fz and Cz during arithmetic verification. **(a)** ERP waveforms at the frontal electrode (Fz). **(b)** ERP waveforms at the central electrode (Cz). Dashed lines represent children with DD, and solid lines represent TD children. Black lines indicate correct proposed-answer trials, and red lines indicate incorrect proposed-answer trials. The N2a, N2b, and P3 measurement windows are indicated.

Grand-average ERP waveforms time-locked to the onset of the proposed answer are shown in **Figure 2**. Omnibus mixed-design ANOVAs were first conducted separately for peak amplitude and peak latency to examine whether the pattern of group differences varied across ERP components and electrodes. Component- and electrode-specific 2 × 2 mixed-design ANOVAs were then examined as follow-up analyses, with statistical interpretation based on Holm-adjusted p values.

#### 3.3.1 Omnibus analyses across ERP components

For peak amplitude, the Group × Component interaction was significant, *F*(1.58, 116.80) = 9.077, *P* < 0.001, ηp² = 0.109. The Group × Component × Electrode interaction was also significant, *F*(1.40, 103.59) = 4.740, *P* = 0.020, ηp² = 0.060. These results indicate that the pattern of group differences in peak amplitude varied across ERP components and electrodes.

For peak latency, the Group × Component interaction was significant, *F*(1.66, 122.63) = 12.150, *P* < 0.001, ηp² = 0.141. The Group × Component × Electrode interaction was also significant, *F*(1.71, 126.26) = 12.266, *P* < 0.001, ηp² = 0.142. Thus, the pattern of group differences in peak latency also varied across ERP components and electrodes.

#### 3.3.2 N2a

For N2a peak amplitude, no significant main effects of Group or Answer Type and no significant Group × Answer Type interactions were observed at Fz or Cz (all unadjusted *P* ≥ 0.065).

For N2a peak latency, the main effect of Group was significant at Fz, *F*(1, 74) = 17.624, unadjusted *P* < 0.001, Holm-adjusted *P* < 0.001, ηp² = 0.192, and at Cz, *F*(1, 74) = 160.423, unadjusted *P* < 0.001, Holm-adjusted *P* < 0.001, ηp² = 0.684. Children with DD showed longer N2a peak latencies than TD children at both electrodes. Neither the main effect of Answer Type nor the Group × Answer Type interaction was significant at Fz or Cz (all unadjusted *P* ≥ 0.384).

#### 3.3.3 N2b

For N2b peak amplitude at Fz, nominal effects were observed for Group, *F*(1, 74) = 4.122, unadjusted *P* = 0.046, ηp² = 0.053, and Answer Type, *F*(1, 74) = 6.902, unadjusted *P* = 0.010, ηp² = 0.085. However, neither effect remained significant after Holm correction (Holm-adjusted *P* = 0.321 and 0.115, respectively). At Cz, the nominal Answer Type effect, *F*(1, 74) = 7.579, unadjusted *P* = 0.007, ηp² = 0.093, also did not survive Holm correction (Holm-adjusted *P* = 0.089). No other peak-amplitude effects were significant.

For N2b peak latency at Fz, the main effect of Group remained significant after Holm correction, *F*(1, 74) = 60.364, unadjusted *P* < 0.001, Holm-adjusted *P* < 0.001, ηp² = 0.449, with children in the DD group showing longer latencies than TD children. At Cz, a nominal Group effect was observed, *F*(1, 74) = 4.946, unadjusted P = 0.029, ηp² = 0.063, but it did not survive Holm correction (Holm-adjusted *P* = 0.234). No Answer Type or Group × Answer Type effects remained significant after correction.

#### 3.3.4 P3

For P3 peak amplitude at Fz, the main effect of Group remained significant after Holm correction, *F*(1, 74) = 15.731, unadjusted *P* < 0.001, Holm-adjusted *P* = 0.002, ηp² = 0.175. Children with DD showed a smaller P3 peak amplitude than TD children. At Cz, a nominal Group × Answer Type interaction was observed, *F*(1, 74) = 5.354, unadjusted *P* = 0.023, ηp² = 0.067, but it did not survive Holm correction (Holm-adjusted P = 0.281). No other peak-amplitude effects were significant.

For P3 peak latency, no significant main effects of Group or Answer Type and no significant Group × Answer Type interactions were found at Fz or Cz (all unadjusted *P* ≥ 0.159).

### 3.4 Supplementary sensitivity analyses

Supplementary 2 × 2 mixed-design ANCOVAs were conducted with the WISC-IV Working Memory Index and Processing Speed Index entered simultaneously as covariates. After adjustment, the Group effects remained significant for N2a latency at Fz, *F*(1, 72) = 17.61, *P* < 0.001, ηp² = 0.196, and Cz, *F*(1, 72) = 159.00, *P* < 0.001, ηp² = 0.688, with longer latencies in the DD group. The Group effect also remained significant for N2b peak latency at Fz, *F*(1, 72) = 58.76, *P* < 0.001, ηp² = 0.449, and for P3 peak amplitude at Fz, *F*(1, 72) = 11.82, *P* = 0.001, ηp² = 0.141. Thus, adjustment for working memory and processing speed did not alter the direction or statistical significance of the four principal Group effects that survived Holm correction in the primary analyses.

### 3.5 Brain–behavior associations

In exploratory analyses across the full sample, N2a peak latency was positively correlated with reaction time during the arithmetic-verification task. At Fz, a significant correlation was observed for correct trials (*r* = 0.261, *P* = 0.023). At Cz, N2a latency was significantly correlated with reaction time for correct trials (*r* = 0.478, *P* < 0.001) and incorrect trials (*r* = 0.459, *P* < 0.001).

When the DD and TD groups were analyzed separately, none of the within-group correlations reached significance (all *P* > 0.05). The full-sample associations should therefore be interpreted cautiously, as they may partly reflect separation between the two groups rather than a consistent linear association within either group. The present findings do not support the use of N2a peak latency as an individual-level predictor.

**Figure 3:**
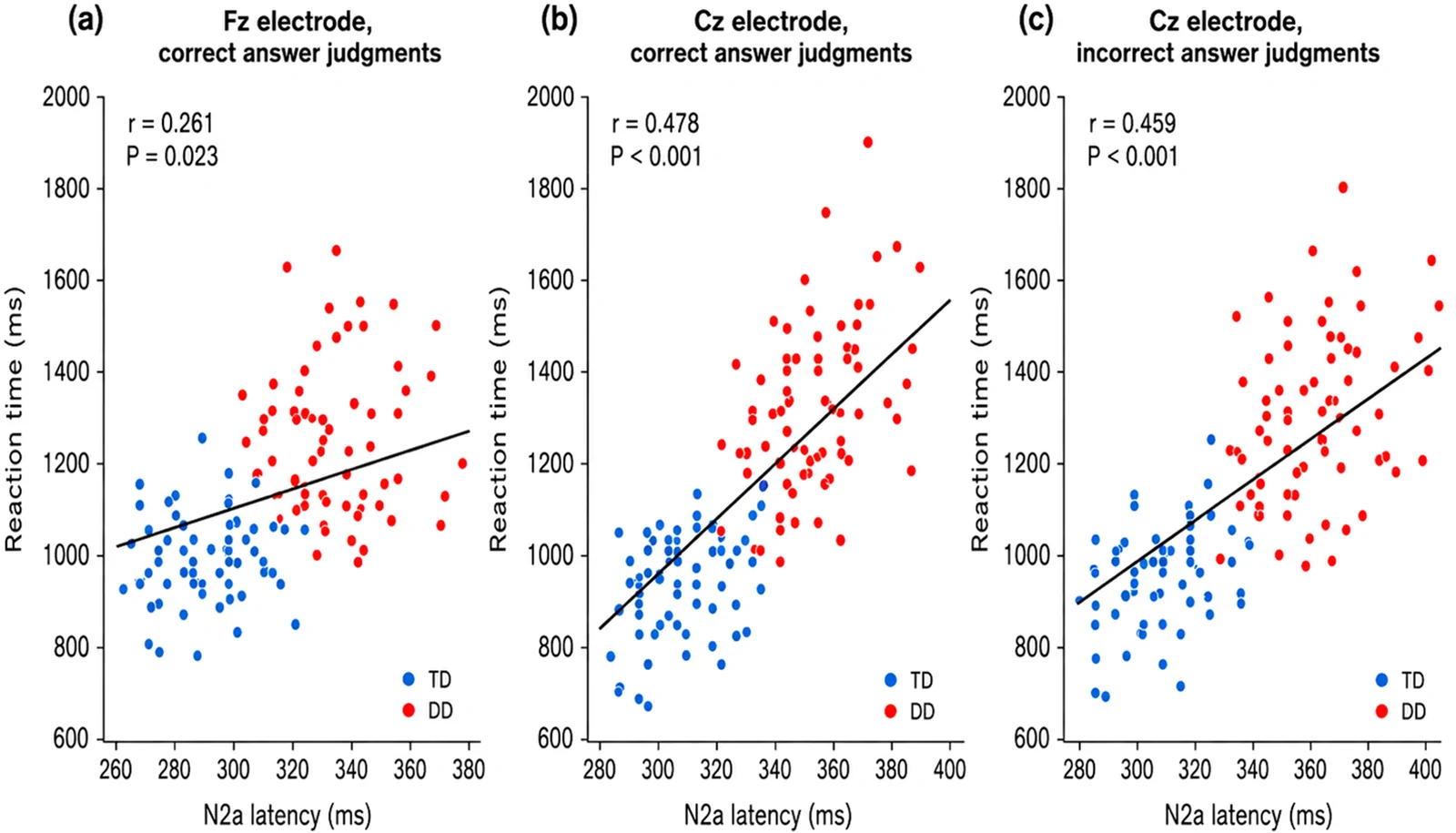
Associations between N2a peak latency and reaction time during arithmetic verification. Scatterplots **(a–c)** show the associations between N2a peak latency (ms) and reaction time (ms) at the frontal (Fz) and central (Cz) electrodes. Blue circles represent TD (typically developing) children, and red circles represent children with DD (developmental dyscalculia). The black line represents the regression across the full sample. Pearson correlation coefficients (r) and P values are provided for each panel.

Scatterplots illustrating the associations between N2a latency and reaction time at Fz and Cz electrodes are shown in **Figure 3**.

## 4 Discussion

This study examined answer-locked neural processing during arithmetic verification in children with DD. Children with DD performed less accurately and more slowly than TD children. After Holm correction, the robust ERP group differences were limited to longer N2a peak latencies at Fz and Cz, longer N2b peak latency at Fz, and smaller P3 peak amplitude at Fz. The omnibus analyses further indicated that the pattern of group differences varied across ERP components and electrodes. These findings characterize altered answer-related neural dynamics during arithmetic verification, although the present task cannot determine whether the differences reflect calculation difficulty, verification-related demands, or their interaction. In supplementary sensitivity analyses, the four principal group effects remained significant after adjustment for the WISC-IV Working Memory and Processing Speed indices, suggesting that these findings were not fully accounted for by individual differences in these cognitive abilities.

The surviving ERP effects were moderate to large in statistical magnitude, with partial eta-squared values ranging from 0.175 to 0.684. However, statistical effect size should not be equated with educational or clinical utility. The present study did not evaluate diagnostic accuracy, test–retest reliability, or prediction of everyday mathematical functioning. Accordingly, the ERP findings should be interpreted as group-level process indicators rather than clinically actionable markers.

The behavioral findings are consistent with number-specific accounts of DD, which emphasize weaknesses in numerical magnitude representation, symbolic number mapping, and arithmetic fact retrieval (1–3). However, arithmetic verification is a composite task. Once the proposed answer appears, the child must compare it with an internally generated result, judge whether the two match, and select a response. This sequence depends on arithmetic result generation or retrieval, working-memory maintenance, answer comparison, and response selection. The present study adds to prior work by providing an answer-locked temporal characterization of group differences during the answer-verification phase, without establishing that these differences are independent of calculation difficulty.

The N2a findings showed the most consistent latency difference during the earlier post-answer period. Children with DD had longer N2a peak latencies at both Fz and Cz, whereas no N2a peak-amplitude effect remained significant. N2-related activity has been associated with mismatch, conflict-related processing, and response selection in previous paradigms (11–13, 26, 27). In the present task, the proposed answer created a comparison point between the externally presented answer and an expected internal result. The later N2a peak indicates altered neural timing during an early post-answer period, but does not identify whether the difference arose from result generation, maintenance, answer comparison, or interactions among these demands.

This interpretation is consistent with evidence that DD is not limited to impaired number representation. Children with DD have been reported to show poorer performance on inhibitory-control and visuospatial working-memory tasks (8, 9). In addition, executive functions are closely related to mathematical development. Inhibition, shifting, and working memory have all been associated with children’s mathematical performance (4–7, 10). The prolonged N2a latency observed here is compatible with this broader profile, but may also reflect difficulty generating or maintaining a reliable expected result before comparison.

The N2b findings provide evidence of a later timing difference at the frontal site. After Holm correction, children with DD showed longer N2b peak latency at Fz, whereas the Group effect on N2b peak amplitude and the Answer Type effects did not remain significant. The prolonged frontal N2b latency indicates altered neural timing during a subsequent post-answer period. However, because the task did not isolate response control, inhibition, or answer comparison, this effect may reflect arithmetic difficulty, maintenance of the expected result, response selection, or interactions among these demands.

The P3 findings indicate a later group difference in answer-related processing. After Holm correction, children with DD showed reduced frontal P3 amplitude, whereas P3 latency did not differ significantly between groups. The amplitude result was consistent with our directional hypothesis. However, the predicted prolongation of P3 latency was not observed, suggesting that the group difference at this stage was expressed primarily in response magnitude rather than peak timing. P3 amplitude is commonly associated with attentional resource allocation, context updating, stimulus evaluation, and decision-related processing (15, 28–32). In the present study, the smaller frontal P3 response indicates altered neural activity during a later post-answer period, but does not identify a specific underlying cognitive process. This amplitude difference co-occurred with slower and less accurate behavioral performance, although the present data do not establish a causal relationship between the neural and behavioral findings.

Taken together, the N2a, N2b, and P3 results indicate group differences during earlier and later periods following proposed-answer onset. The omnibus analyses further showed that the pattern of group differences varied across ERP components and electrodes for both peak amplitude and peak latency. However, these interactions do not establish differences in the severity of impairment across distinct cognitive stages. Because arithmetic calculation, maintenance of the expected result, answer comparison, and response selection were not experimentally separated, the findings cannot establish a sequential disruption of cognitive control.

This pattern also fits a multicomponent view of DD. Mathematical performance depends on several partially overlapping systems, including numerical representation, working memory, attention, and strategy use (33). Neuroimaging studies have similarly shown that arithmetic relies not only on parietal numerical regions, but also on frontal and attentional networks that support working memory and goal-directed task performance (34, 35). Studies of DD have reported atypical activation and connectivity within these networks, including weaker activation during approximate calculation and abnormal spatial working-memory networks (36–38). The present ERP findings add temporal evidence by showing when verification-related group differences emerge after the proposed answer appears.

The brain–behavior correlations provide complementary but cautious evidence. Across the full sample, longer N2a peak latencies were associated with slower reaction times during arithmetic verification, particularly at Cz. However, none of these associations was significant when the DD and TD groups were analyzed separately. The full-sample correlations may therefore partly reflect separation between the two groups rather than a consistent linear association within either group. Accordingly, the present findings do not establish that delayed N2a activity caused slower behavioral responses, and N2a peak latency should not be interpreted as an individual-level predictor.

The findings may have implications for future process-oriented assessment and intervention research. Interventions for DD typically focus on number knowledge, arithmetic fact retrieval, and calculation strategies. The present results raise the hypothesis that future studies could examine whether training that includes answer checking or verification is accompanied by changes in answer-locked ERP responses. Previous work has shown that targeted mathematical training can alter behavioral performance and brain function in children with mathematical learning difficulties (39, 40). However, the present cross-sectional findings do not demonstrate that verification-focused training would be effective, identify a causal intervention target, or support the use of these ERP measures as diagnostic biomarkers or individual-level predictors.

Several limitations should be acknowledged. First, arithmetic verification is a composite task, and the design did not include an independent calculation-only or non-arithmetic control condition, nor did it manipulate the numerical distance or plausibility of incorrect answers. The correct–incorrect contrast therefore cannot isolate inhibitory load. The observed group differences may reflect arithmetic slowing, difficulty generating or maintaining an expected result, later comparison and decision processes, or interactions among these demands. Second, although all participants scored within the non-clinical range on general anxiety and depression measures, mathematics-specific anxiety was not assessed using a dedicated instrument and may have influenced arithmetic performance and answer-related neural responses. Third, the Z ≤ −1.0 mathematical-performance cutoff was less restrictive than that used in some DD studies and may have captured a relatively broad phenotype, although it was applied as part of a multistep identification procedure rather than as the sole criterion for DD. Fourth, although no participant was excluded solely because of the 80% trial-retention criterion, requiring a high proportion of valid trials may still limit generalizability to children who can remain sufficiently still and engaged during EEG recording. Children with more severe task-engagement or movement difficulties may therefore be underrepresented in EEG studies using this type of data-quality threshold. Fifth, the analysis focused on Fz and Cz; broader scalp analyses, topographic mapping, or source estimation could provide a more complete account of the underlying neural activity. Sixth, the sample size may have limited the detection of within-group brain–behavior associations. Seventh, the 8–12-year age range spans substantial developmental change, and the cross-sectional design does not allow conclusions about developmental trajectories. Finally, the present analyses combined arithmetic operations, and future studies should examine whether answer-locked ERP responses vary by operation type using designs adequately powered to test Answer Type × Operation interactions.

In conclusion, children with DD showed less accurate and slower performance during arithmetic verification, together with robust answer-locked ERP differences after correction for multiple testing. These differences included longer N2a peak latencies at Fz and Cz, longer N2b peak latency at Fz, and smaller P3 peak amplitude at Fz. The omnibus analyses further indicated that the pattern of group differences varied across ERP components and electrodes. These findings characterize altered neural timing and response magnitude following proposed-answer onset. However, because arithmetic result generation, maintenance of the expected result, answer comparison, and response selection were not experimentally separated, the findings do not establish a sequential disruption of distinct cognitive stages or an isolated deficit in inhibition or cognitive control.

## Data Availability

The raw data supporting the conclusions of this article will be made available by the authors, without undue reservation.
